# Peak Torque and Rate of Torque Development Influence on Repeated Maximal Exercise Performance: Contractile and Neural Contributions

**DOI:** 10.1371/journal.pone.0119719

**Published:** 2015-04-22

**Authors:** Baptiste Morel, David M. Rouffet, Damien Saboul, Samuel Rota, Michel Clémençon, Christophe A. Hautier

**Affiliations:** 1 Center of Research and Innovation on Sport, University of Lyon 1, Villeurbanne, France; 2 Institute of Sport, Exercise and Active Living, Victoria University, Melbourne, Australia; 3 Almerys, Clermont-Ferrand, France; East Tennessee State University, UNITED STATES

## Abstract

Rapid force production is critical to improve performance and prevent injuries. However, changes in rate of force/torque development caused by the repetition of maximal contractions have received little attention. The aim of this study was to determine the relative influence of rate of torque development (RTD) and peak torque (T_peak_) on the overall performance (i.e. mean torque, T_mean_) decrease during repeated maximal contractions and to investigate the contribution of contractile and neural mechanisms to the alteration of the various mechanical variables. Eleven well-trained men performed 20 sets of 6-s isokinetic maximal knee extensions at 240°·s^-1^, beginning every 30 seconds. RTD, T_peak_ and T_mean_ as well as the Rate of EMG Rise (RER), peak EMG (EMG_peak_) and mean EMG (EMG_mean_) of the *vastus lateralis* were monitored for each contraction. A wavelet transform was also performed on raw EMG signal for instant mean frequency (i*f_mean_*) calculation. A neuromuscular testing procedure was carried out before and immediately after the fatiguing protocol including evoked RTD (eRTD) and maximal evoked torque (eT_peak_) induced by high frequency doublet (100 Hz). T_mean_ decrease was correlated to RTD and T_peak_ decrease (R²=0.62; p<0.001; respectively β=0.62 and β=0.19). RER, eRTD and initial i*f_mean_* (0-225 ms) decreased after 20 sets (respectively -21.1±14.1, -25±13%, and ~20%). RTD decrease was correlated to RER decrease (R²=0.36; p<0.05). The eT_peak_ decreased significantly after 20 sets (24±5%; p<0.05) contrary to EMG_peak_ (-3.2±19.5 %; p=0.71). Our results show that reductions of RTD explained part of the alterations of the overall performance during repeated moderate velocity maximal exercise. The reductions of RTD were associated to an impairment of the ability of the central nervous system to maximally activate the muscle in the first milliseconds of the contraction.

## Introduction

Fatigue can be defined as a reduction in the force generating capacity of the neuromuscular system, regardless of the level of force required [1]. During exercises of maximal intensity, fatigue can result into declines of force [2, 3], contraction velocity [4, 5] or power [4, 6, 7]. In recent years, numerous studies have investigated the mechanisms at the origin of fatigue during intermittent maximal isokinetic contractions interspersed by short recovery periods. Most studies have investigated fatigue-induced impairments of human performances by focusing on reductions of the average levels and/or peak levels of force, diminutions of peak movement velocity, or changes in the curvature of the force-velocity relationships. However, the effect of fatigue on the ability to produce force rapidly has received less attention despite its importance for the production of many movements. Indeed, rate of force development (RFD) is a key factor of performance during movements characterized by reduced contractions times (<250ms) such as sprinting, jumping or kicking [8]. Furthermore, maintaining the ability of producing high RFD values is important to limit the risks of injury following mechanical perturbation [9]. The understanding of how rate of force development (RFD) is impaired during intermittent maximal exercise is decisive for a better comprehension of athletic performance and injury risk.

The origin of fatigue following repeated maximal isokinetic contractions has been previously investigated. Following a series of contractions performed at moderate velocity, it has been shown that evoked peak twitch responses are substantially reduced (by 25 to 75%) [2, 5, 10]. Conversely, investigation of changes in voluntary activation using the twitch interpolation technique only revealed slight reductions (by 3 to 5%) [2, 5, 10] of the ability of the central nervous system to maximally activate the muscles following repeated maximal contractions. Because of the relatively long duration of maximal isometric contractions, one can wonder if the twitch interpolation technique can be used to identify manifestations of central fatigue causing reductions of rapid force production. Recent studies showed that, during simulated team sport matches, the RFD decrease was associated with a decrease in EMG signal during the early phase of the contraction (0–100 ms). Therefore, fatigue induced by repeated maximal contractions manifests by a reduction of RFD that might be due to an alteration of the ability to maximally and rapidly activate the muscles [11, 12]. The contractile component of the RFD, estimated during evoked twitch, has also been evidenced to be altered following a fatiguing protocol of repeated maximal contractions [13]. However, no study has investigated the link between fatigue-induced reductions of RFD and changes in the EMG signals during the early part of the contraction after repetition of a series of maximal contractions performed at moderate velocities.

The first aim of this study was to evaluate the relative contribution of reductions in the rate of torque development and diminutions of the peak torque on the decrease of the average torque measured during repeated maximal contractions performed at a moderate velocity (240 degrees/s). The second aim was to investigate the link between fatigue-induced reductions of RTD and changes in the contractile (twitch) and neural (EMG) responses. It was hypothesized that i) reductions of RTD have a substantial impact on the decrease of overall performance produced during repeated maximal contractions performed at moderate velocities and ii) diminutions of RTD would be associated to alterations of the characteristics of the evoked torque and the EMG signal during the initial phase of the contraction.

## Materials and Methods

### Subjects

Eleven males volunteered to participate in this study (mean ± SD; age: 22.7 ± 1.9 year; mass: 87.6 ± 8.6 kg; height: 1.82 ± 0.06 m; training volume: 11.1 ± 3.1 h.wk^-1^). All participants regularly performed maximal efforts involving their lower limb muscles. Written informed consent was obtained from the subjects, and the study was conducted according to the declaration of Helsinki. Approval for the project was obtained from the Lyon 1 University ethics committee on human experimentation.

### Experimental design

**Fig 1:**
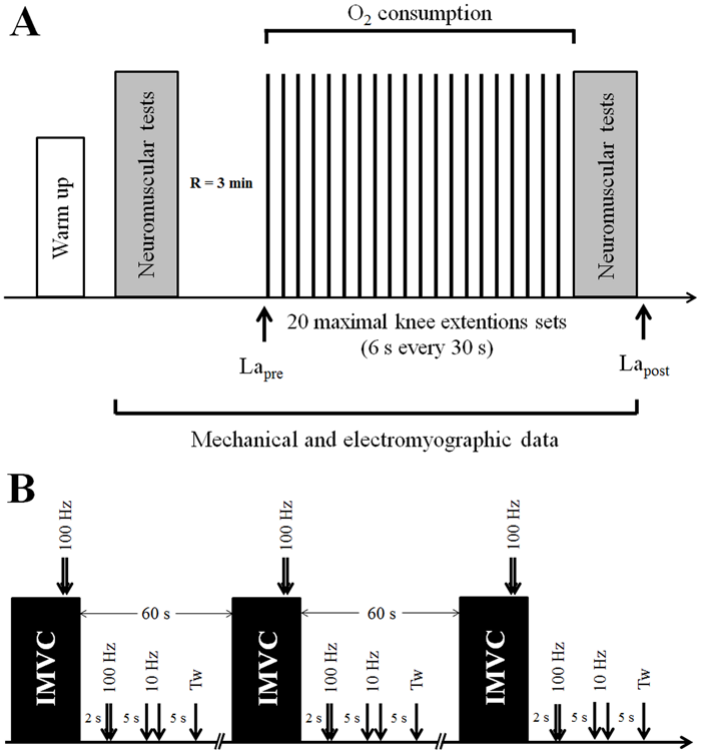
Experimental design (A) and neuromuscular testing procedure (B). Arrows indicate the timing of (A) blood micro sampling for lactate analysis (La_pre_ and La_post_) and (B) motor nerve stimulation. Three types of stimulation were performed: doublet stimulation at 100 Hz, doublet stimulation at 10 Hz and single twitch (Tw). Stimulations were delivered during the isometric maximal voluntary contraction (IMVC) and on the relaxed potentiated muscle (baseline).

First, the subjects participated in a familiarization session to become accustomed to the testing procedures. At the start of the experimental session, participants completed a standardized warm-up protocol that consisted of a 10-min cycling exercise completed at 1 W·kg^-1^ of body mass immediately followed by a series of knee extensions (standardized order, perceived intensity from 70% at the beginning to 100% at the end of the warm-up, recovery between sets = 45 s: 8 × 240°·s^-1^; 6 × 180°·s^-1^; 4 × 90°·s^-1^; 2 × 30°·s^-1^; 2 × 0°·s^-1^). Following a 5-min recovery period, participants completed a pre-exercise neuromuscular testing before completing the exercise protocol (i.e. repeated maximal knee extensions). Immediately after completion of the exercise protocol, participants performed a post-exercise neuromuscular testing (see Fig 1).

The exercise protocol consisted of total of 160 isokinetic maximal knee extensions that were performed at 240°·s^-1^, with the knee joint angle changing from 105° to 15° (0° corresponding to full knee extension) so that each contraction lasted ~375ms. The 160 contractions were performed in the following manner: participants completed 20 sets of eight repetitions each (work/rest ratio = 1 during each set) and were given 24s recovery between each set (see Fig 1). At the end of each maximal knee extension, an experimenter manually pushed the arm to its initial position while the participants were instructed to relax their knee flexors during this phase. Participants were instructed to “push as hard and as fast as possible” during each contraction and were provided with a real-time visual feedback allowing them to monitor the torque produced during each maximal knee extension. Researchers provide participants with strong verbal encouragements during the whole duration of the exercise. Immediately after completion of the 160 maximal knee extensions, participants completed the same the neuromuscular testing (i.e. post-exercise neuromuscular testing).

For the neuromuscular testing (organized pre- and post-exercise) participant were instructed to perform three isometric maximal voluntary contractions (IMVC) of 4-s duration each interspersed by 60-s rest with the knee positioned at 90°. During each IMVC, the femoral nerve was electrically stimulated. First a high-frequency doublet stimulus (100 Hz) was delivered on the torque plateau (identified using a real-time visual display of the torque signal) while the subjects were instructed to prolong their IMVC for about 2 s after the stimulation. After each IMVC, participants were instructed to relax their muscles. During this period, one high frequency doublet stimulation (100 Hz) was applied 2 s after the end of the IMVC followed by low frequency (10 Hz) doublet stimulation 5 s after the first stimulation and a single twitch was applied 5 s later. The ratio between Db_10_ and eT_peak_ (Db_10:100_) was also calculated to investigate if there was any predominance of low or high frequency fatigue, respectively associated to Ca^2+^ release-reuptake or sarcolemmal disruption [14]. For both pre- and post-exercise neuromuscular testing, average values of eT_peak_, Db_10:100_ and eRTD were calculated over the three measurements obtained as no changes were observed between measurements during the post-exercise testing. Peripheral nerve stimulations were delivered using an electrical stimulator (Digitimer DS7AH, Hertfordshire, UK) and consisted of a square wave stimuli (200 μs duration and 400 V) delivered by at supra-maximal intensity (i.e. 150% of the stimulation intensity inducing maximal mechanical and electrical response). All electrical stimulations were delivered by percutaneously stimulating the femoral nerve using a self-adhesive cathode (10-mm diameter, Ag-AgCl, Contrôle Graphique Medical, Brie-Comte-Robert, France) that was pressed manually over the femoral triangle while the anode (a 10 × 5 cm self-adhesive stimulation electrode, Medicompex SA, Ecublens, Switzerland) was positioned in the gluteal fold.

### Measurements and data analysis

#### Torque

**Fig 2:**
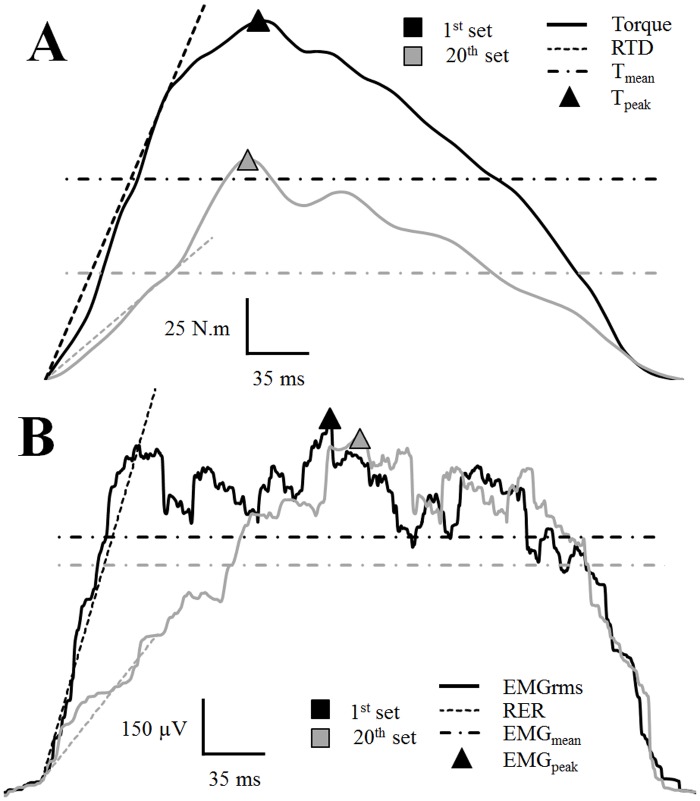
Torque and EMG signals recorded at the start and end of the exercise protocol. A- Illustration of typical changes in average torque traces recorded during set 1 (black line) and set 20 (grey line) and associated changes in the torque variables extracted: T_mean_ (dash-dotted lines), T_peak_ (triangles) and RTD (dotted lines). B—Illustration of the typical changes in average EMG RMS signal of VL muscle recorded during set 1 (black line) and set 20 (grey line) and associated changes in the EMG variables extracted: EMG_mean_ (dash-dotted lines), EMG_peak_ (triangles) and RER (dotted lines).

The torque was measured by the isokinetic dynamometer (Biodex 3, Biodex, Shirley, NY) during the neuromuscular testing (pre and post-exercise) and the exercise protocol (maximal repeated knee extensions). The subjects were seated with their hip joint angles set at 80° (0° is full extension) and their chest and working leg tightly fixed against the chair in order to limit lateral and frontal displacement. They were also asked to cross their arms over the chest during the voluntary contractions to limit the involvement of peripheral muscles. For each contraction, both torque and angular velocity signals were recorded (sampling rate: 100 Hz). The isokinetic dynamometer used an automated gravity correction. During the exercise protocol, the following variables were extracted for each contraction (n = 160): time required by the subjects to reach the target angular velocity (240°.s^-1^), average torque (T_mean_), peak torque (T_peak_), and rate of torque development (RTD). RTD values represent the average slope of the torque vs. time curve between 0 and 75ms relative to the contraction onset (Fig 2A).

During the neuromuscular testing (both pre- and post-exercise), IMVC was calculated as the maximal torque value obtained over a 500-ms period preceding the superimposed twitch. The following equation was used to calculate voluntary activation during neuromuscular testing organized pre- and post-exercise (Eq 1):

$$VA\;\left(\%\right)\;=\;\left(1-\frac{superimposed\;twitch}{potentiated\;resting\;twitch}\right)\times 100$$ (1)

For the pre-exercise neuromuscular testing, voluntary activation (VA) was calculated from the superimposed twitch obtained during the highest IMVC (i.e. maximal value obtained from the three contractions). For the post-neuromuscular testing, VA was calculated from the superimposed twitch obtained during the first IMVC in order to avoid any recovery between contractions 1 and 3. Then the torque signal was processed to extract the maximal evoked torque (eT_peak_) and the maximal evoked rate of twitch tension development (eRTD) induced by the high frequency doublet stimulation (100 Hz); as well as the maximal evoked torque (Db_10_) induced by low the frequency doublet stimulation (10 Hz). The ratio between Db10 and eT_peak_ (Db_10:100_) was also calculated to investigate if there was any predominance of low or high frequency fatigue, respectively associated to Ca^2+^ release-reuptake or sarcolemmal disruption [14]. For both pre- and post-exercise neuromuscular testing, average values of eT_peak_, Db_10:100_ and eRTD were calculated over the three measurements obtained as no changes were observed between measurements during the post-exercise testing.

During each contraction performed during the repeated knee extension protocol, the following variables were extracted: average torque (T_mean_), peak torque (T_peak_) rate of torque development (RTD) that was calculated as the average slope of the torque—time curve between 0 and 75 ms relative to the contraction onset. Then, the values obtained for each variable were expressed as a percentage of the maximal values obtained and averaged over 4 set (Fig 2A).

#### Electromyography

EMG activity of the vastus lateralis (VL) muscle of the dominant leg was recorded using surface electrodes (EMG Triode, nickel-plated brass, electrode diameter = 1 cm, inter-electrode distance = 2 cm, Thought Technology, Montreal, Canada). EMG signals were sampled at 2,048Hz using the Flexcomp Infiniti system (Thought Technology, Montreal, Canada). The Flexcomp Infiniti system had an input impedance and common mode rejection ratio of 2 MΩ and >110 dB, respectively. The EMG electrodes were positioned according to SENIAM recommendations [15]. The skin was shaved and cleaned with alcohol before placing electrodes to improve the contact between skin and electrode and to reduce skin impedance (<2 kΩ). The electrodes were placed on the muscle belly and positioned longitudinally to the muscle fibers.

Raw EMGs were filtered (Butterworth order 2, bandpass 10–500 Hz) and amplified (gain = 500) before calculating root mean squared values (RMS) with a 50-ms moving rectangular window (Origin Pro 8.1, OriginLab, Northampton, USA). M-wave peak to peak amplitude (M_max_) was calculated as the voltage difference between the two extreme points of the electromyographic response to optimal electrical stimulation (i.e. M-wave). For each contraction, the following EMG variables were extracted: average EMG (EMG_mean_), peak EMG (EMG_peak_) and rate of EMG increase (RER). RER values represent the average slope of the EMG—time curve between 0 and 75ms relative to the activation onset, since a decrease in EMG signal amplitude typically occurred after 80–100ms [8] (Fig 2B). Average values of the different EMG variables were then calculated for five 20% intervals (i.e. including 32 contractions each) of the exercise protocol (see Fig 1A) and were normalized to the maximal values measured during one of the 160 maximal knee contractions. Additionally, average values were calculated for all EMG variables over the 8 first and last contractions performed and the values were normalized to M_max_ values obtained pre- and post-fatigue, respectively (i.e. nEMG_mean_, nEMG_peak_ and nRER).

A wavelet transform (WT) was made on raw EMG signals for each contraction of the first and the twentieth set. The computation of the WT was made with MATLAB software (R2010a, The MathWorks Inc., Natick, MA, USA) based on the algorithm of Torrence and Compo [16] available at http://paos.colorado.edu/research/wavelets, and modified by Frère, Göpfert, Slawinski and Tourny-Chollet [17] to obtain a set of wavelets similar to the one defined by von Tscharner [18]. The EMG signal was transformed by a wavelet bank following a second-degree polynomial scale function, according to Eq 2:

$$F_{aj}\;=\;\left(\frac{1}{scale}\right)\;\times \;{\left(j+q\right)}^{r}$$ (2)

where *F*_*aj*_ is the center frequency of wavelet j (Hz). For this study, the factor scale = 0.3 in order to define a range of frequencies provided by different wavelets, while factors q (1.45) and r (1.959) optimized the spacing between the wavelets [18]. The mother wavelet used for WT was a ‘Morlet’ wavelet. The instantaneous mean frequency (i*f*_*mean*_) was computed by the relation (Eq 3):

$$ifmean\;=\;\frac{\left(\sum_{j\;=\;0}^{10}F_{aj}\times \;\left(\sum p_{j}\right)\right)}{\left(\sum_{j\;=\;0}^{10}\;\left(\sum p_{j}\right)\right)}$$ (3)

The intensity (Σ*p*_*j*_) for each wavelet j was calculated and corresponded to the power of the signal within the center frequencies of these two wavelet sets [19]. The mean i*fmean* was considered for each 75 ms interval of the contraction (i.e., 0–75, 75–150, 150–225, 225–300 and 300–375 ms).

#### Gas exchange

During the exercise protocol (maximal knee extensions), oxygen consumption (VO_2_) was measured using a mobile gas analyzer (MetaMax 3b; Cortex Biophysik, Leipzig, Germany). The analyzer was warmed up and calibrated according to the manufacturer’s instructions before each measurement. Average VO_2_ values were calculated over 30 s intervals (i.e. 8 contractions + resting period duration). Non-linear regression techniques were used to model changes in VO2 over the whole duration of the exercise protocol. The data were well fitted by a mono-exponential function allowing us to use the asymptote of the function to calculate O_2_ consumption at a steady state.

#### Blood lactate

A micro blood sample was taken from the fingertip and analyzed for blood lactate concentration [La^-^] using a Lactate Pro (LT-1710, Arkray, Japan) portable analyzer. [La^-^] was measured just after completion of the neuromuscular testing organized immediately prior and after the repeated knee extension exercise (i.e. 3 min after the end of the exercise fatiguing protocol). Blood lactate accumulation was obtained by calculating the variation between [La^-^] values measured pre- and post-exercise [20].

### Statistical analysis

All data were analyzed with Statistica 8.0 Software (StatSoft Inc., Tulsa, OK, USA) and were expressed as means ± SD. The normality of the error distribution was examined with the Lilliefors test. Homogeneity of variance was verified using Levene's test. With the assumption of normality and homogeneity of variance confirmed, a one-way analysis of variance (ANOVA) with repeated measures (set) was employed on i*f*_*mean*_, T_mean_, T_peak_, RTD, EMG_mean_, EMG_peak_, and RER. A one-way analysis of variance (ANOVA) with repeated measures (pre- vs. post-fatigue or 1^st^ vs 20^th^ set) was also employed on each pre- vs. post- variable (i.e. IMVC torque, VA, eT_peak_ Db_10:100_, eRTD, M_max_, blood lactate accumulation) and 1^st^ vs 20^th^ set (i.e. T_mean_, T_peak_, RTD, nEMG_mean_, nEMG_peak_, nRER). When a significant interaction was revealed, Tukey’s post hoc test was used to specify where the difference occurred. Forward stepwise multiple linear regression analyses were performed to verify the influence of RTD and T_peak_ on T_mean_ as well as RER and EMG_peak_ on EMG_mean_. If one of the predictor variable did not improved the R^2^, was not significant or correlated to the other one, a simple linear regression was performed. A simple linear regression was used to verify the influence of RER on RTD. The alpha level for statistical significance was set at p<0.05.

## Results

### Torque and EMG changes during the exercise protocol

**Fig 3:**
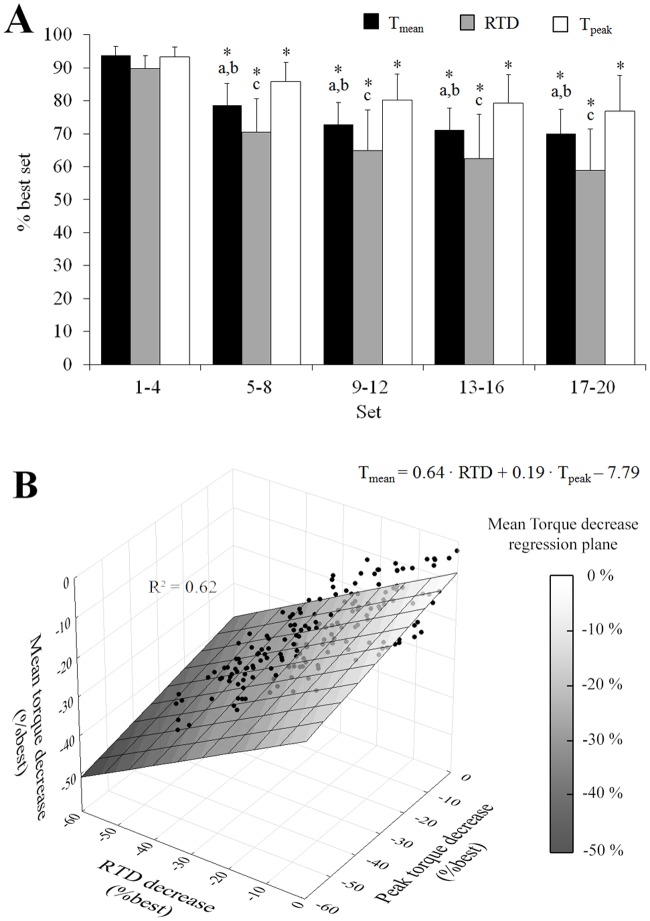
Variations of the torque variables during the exercise protocol. Mean torque (T_mean_), peak torque (T_peak_) and rate of torque development (RTD) decrease over the 20 sets (A) as well as the multiple linear regression of T_mean_ predicted by T_peak_ and RTD (B) is presented. Data are mean ± SD for four sets. Significant difference from set 1–4: * <0.05. Significant difference between T_mean_ and RTD from the same sets: a; Significant difference between T_mean_ and T_peak_ from the same sets: b; Significant difference between RTD and T_peak_ from the same sets: c.

The time to reach the target velocity (57 ± 8 ms vs. 58 ± 10 ms; p>0.05) as well as the time to reach T_peak_ (161 ± 32 ms vs. 156 ± 12 ms, p>0.05) did not vary between the first and last set of contractions. Compared to the sets 1–4, T_mean_, T_peak_ and RTD consistently decreased from the set 5–8 (p<0.05). Larger reductions were observed for RTD variable compared to T_mean_ (p<0.05) and T_peak_ decrease (p<0.001) (Fig 3A). The multiple regression analysis showed that T_mean_ decrease was explained by RTD decrease and T_peak_ decrease (R^2^ = 0.62; p<0.001). Furthermore, the T_mean_ decrease was more influenced (p<0.01) by the RTD decrease (β = 0.64) than T_peak_ decrease (β = 0.19) (Fig 3B).

**Fig 4:**
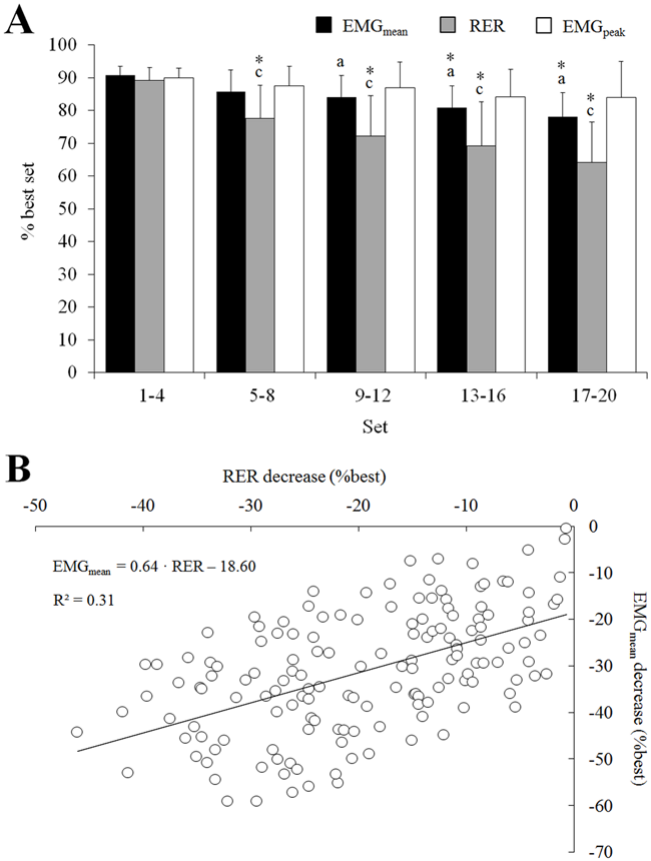
Changes in the EMG variables during the exercise protocol. Mean EMG_rms_ (EMG_mean_), peak EMG_rms_ (EMG_peak_) and rate of EMG rise (RER) decrease over the 20 sets (A) as well as the simple linear regression of EMG_mean_ predicted by RER (B) is presented. Data are mean ± SD for four sets. Significant difference from set 1–4: * <0.05. Significant difference between EMG_mean_ and RER from the same sets: a; Significant difference between EMG_mean_ and EMG_peak_ from the same sets: b; Significant difference between RER and EMG_peak_ from the same sets: c.

**Fig 5:**
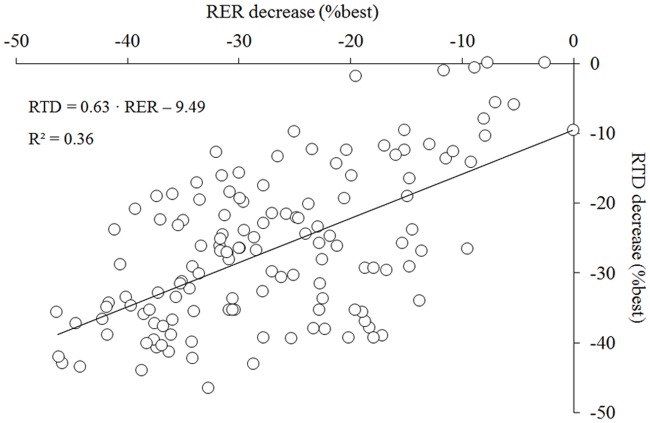
Relationship between changes in rate of EMG rise and variations of rate of torque development during the exercise protocol. The simple linear regression of the rate of torque development (RTD) predicted by the rate of rate of EMG rise (RER) is presented.

Analysis of the EMG signals, showed that RER consistently decreased between the five groups of contraction sets considered (p<0.05) whereas a reduction in EMG_mean_ values was observed only from 13–16 sets (p<0.05). The reduction in RER was larger compared to the decrease in EMG_mean_ (p<0.001) (Fig 4A). The time to reach EMG_peak_ remained unchanged during the exercise protocol (1^st^ set: 228 ± 81 ms; 20^th^ set: 234 ± 82 ms; p>0.05). Changes in EMG_mean_ were best explained by variations in RER (R^2^ = 0.31; p<0.001; Fig 4B). A positive correlation has also been evidenced between RTD and RER (R^2^ = 0.36; p<0.001) (Fig 5).

### Torque and EMG differences between the first and 20^th^ set of contractions

**Fig 6:**
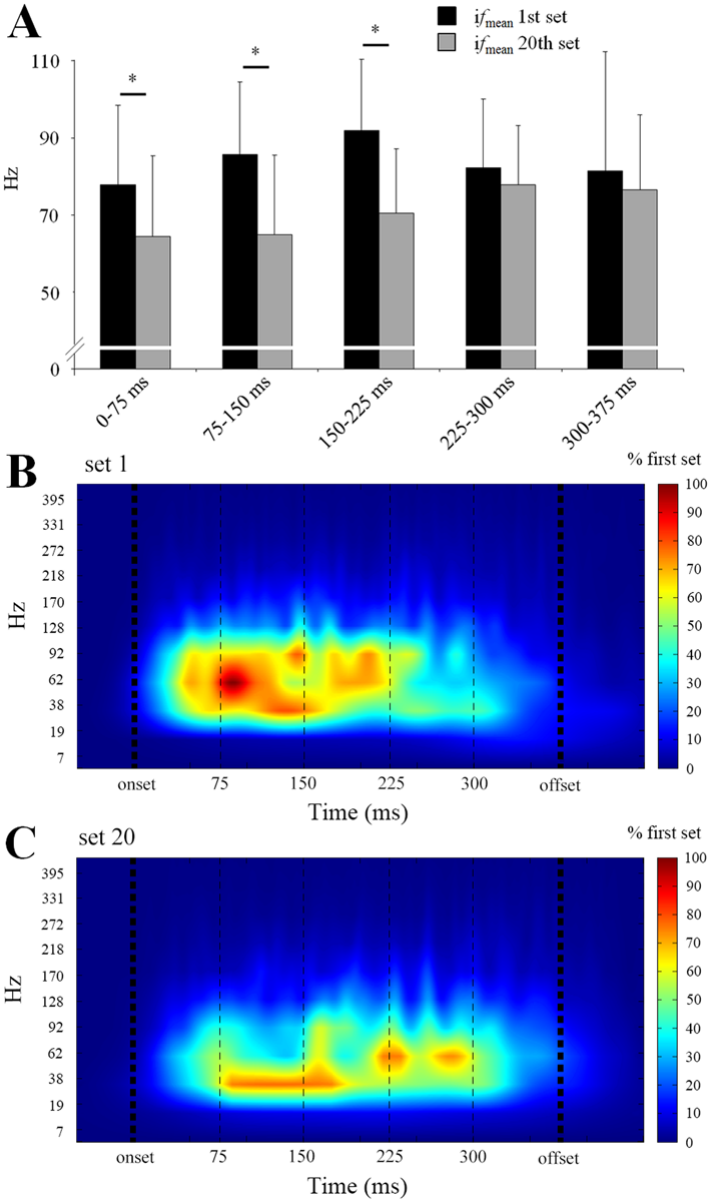
Changes in time-frequency EMG parameters during the exercise protocol. Instant mean power frequency (i*fmean*) during the first (black) and the twentieth (grey) set within each portion (75 ms) of the contraction is presented on panel A (A). Significant differences first *vs*. twentieth set: *: p<0.05, **: p<0.01, ***: p<0.001. Wavelet transform representation of the *vastus lateralis* EMG signal of one contraction is presented for the first (B) and the twentieth (C) set. The intensity patterns are indicated on a colour scale (0–100% of the first set), with red corresponding to higher intensities. The frequency content is expressed in pseudo-frequencies (Hz) calculated from wavelet transform. Data are mean of the eight contractions of the eleven subjects for the first and the twentieth set.

As shown in Fig 6, reductions in all torque variables were observed during the last set of the exercise protocol: T_mean_ = 94.0 ± 18.5 vs 59.8 ± 9.5 N.m (p<0.001), T_peak_ = 172.8 ± 27.5 vs. 134.8 ± 31.7 N.m (p<0.001) and RTD = 1069 ± 352 vs. 561 ± 174 N.m.s^-1^ (p<0.001).

**Fig 7:**
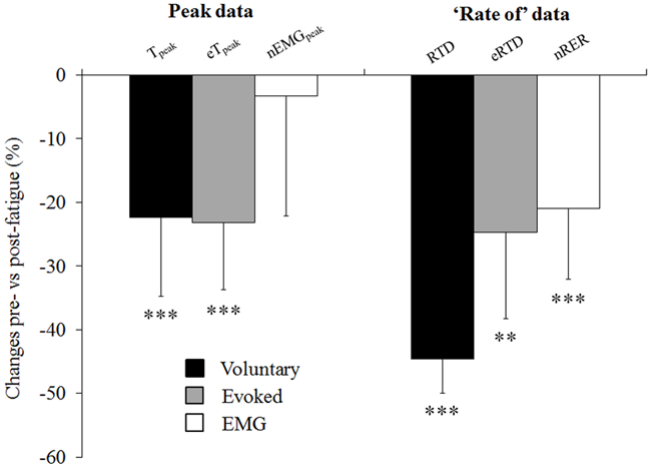
Changes pre- *vs*. post-fatigue for peak and ‘rate of’ data. T_peak_: voluntary peak torque; eT_peak_: evoked peak torque; nEMG_peak_: peak EMG_rms_ normalized with M_max_; RTD: voluntary rate of torque development; eRTD: evoked rate of torque development; nRER: rate of EMG Rise normalized with M_max_. Data are mean ±SD. Significant changes post- vs pre-fatigue: *: p<0.05, **: p<0.01, ***: p<0.001.

Analysis of the EMG signals revealed that changes in most EMG variables were observed during the last set of the exercise protocol: nEMG_mean_ = 0.110 ± 0.051 vs. 0.089 ± 0.029 a.u. (p = 0.04); nRER = 1.42 ± 0.63 vs. 1.09 ± 0.40 a.u. (p<0.001). The i*f*_*mean*_ was significantly lower at the end compared to the beginning of the fatiguing exercise, only in the first milliseconds of the contraction, before EMG_peak_ was reached, i.e. 0–75, 75–150 and 150–225 ms (p<0.001) (Fig 7A). However, no changes in the nEMG_peak_ were observed at the end of the exercise protocol (0.177 ± 0.082 a.u. vs. 0.169 ± 0.048 a.u.; p = 0.71; Fig 6).

### Neuromuscular parameters changes between pre- and post-fatigue tests

IMVC was reduced after the exercise (-32.6 ± 6.3%) (Table 1) and the high frequency doublet (100 Hz) induced both a lower peak torque (eT_peak_: 120.6 ± 19.7 N·m vs. 90.4 ± 12.7 N·m; p<0.001) and a lower rate of torque development (eRTD: 3463 ± 962 N·m·s^-1^ vs. 2559 ± 702 N·m·s^-1^; p = 0.002) (Fig 6). Db_10:100_ was also reduced (-26.8 ± 10.8%) (Table 1). The M-wave peak to peak amplitude (M_max_) did not change from pre- to post-fatigue (-4.9 ± 20.5%; p = 0.62) as well as the VA (-4.3 ±1.6%, p = 0.08) (Table 1).

**Table 1:** Neuromuscular testing data obtained pre- and immediately post-exercise.

	Pre-fatigue	Post-fatigue
**IMVC** (N·m)	320 ± 101	215 ± 62 ***
**VA** (%)	96.4 ± 4.0	92.3 ± 4.9
**eT**_**peak**_ (N·m)	121 ± 21	92 ± 13 ***
**Db**_**10:100**_ (a.u.)	0.89 ± 0.11	0.64 ± 0.08 ***
**M**_**max**_ (mV)	6.34 ± 2.87	6.13 ± 3.36

Values are mean ± SD; IMVC: isometric maximal voluntary torque; VA: voluntary activation; eT_peak_: Peak torque during 100 Hz doublet stimulation; Db_10:100_: ratio between peak torque at 10 Hz and 100 Hz doublet stimulation; M_max_: M-wave peak to peak amplitude.

***: Significant difference with pre-fatigue (p<0.001).

### Metabolic data

The mean plateau of O_2_ consumption estimated from the asymptote was 23.7 ± 6.4 mL·min^-1^·kg^-1^.and the blood lactate accumulation attained 5.7 ± 4.0 mmol·L^-1^ (p<0.001).

## Discussion

The results of this study indicate that the large reductions of average torque measured during maximal intermittent contractions performed at 240 degrees/s were strongly associated with decreases of RTD values. Reductions of both EMG and evoked torque characteristics were observed during the first milliseconds of the contraction, indicating that central and peripheral mechanisms could explain the fatigue-induced reductions of RTD resulting from the repetition of maximal contractions at moderate velocity. Maximal intermittent contractions performed at moderate velocity also led to some reductions of peak torque values that had a smaller impact on the overall performance and were mainly associated to manifestations of peripheral fatigue.

### Manifestation of neuromuscular fatigue during the repeated knee extensions

The relative reductions of mean torque (-31.4 ± 9.6%) and peak torque (-22.3 ± 12.4%) observed in the present study (Fig 3A) are slightly higher than those previously reported for exercise protocols that used comparable contraction velocity, contraction number and duty cycle [2, 21]. One can assume that the participants of our study may have been able to reach higher levels fatigue because they were accustomed to the repetition of maximal exercises [22]. A major finding of this study was the large reduction of RTD observed after 160 maximal contractions (-45.1 ± 14.5%, p<0.05; Fig 3A). This result demonstrates that an important manifestation of fatigue during repeated maximal contractions performed at moderate velocity consists into a substantial alteration of rapid force production. This result is in line with the reductions of RTD previously reported following the repetition of multi-joint movements performed at maximal intensity, such as sprint running and sprint cycling [11, 12, 23]. Our results indicate that it is very important that researchers investigate the effect of fatigue on human performance by carefully selecting the mechanical variable(s); e.g. average force, peak force, RTD [24]. Importantly, our results show that the reduction in rate of torque development had a greater effect on mean torque decrease (β = 0.62) compared to the diminution of peak torque (β = 0.19) during maximal repeated contractions completed at a moderate velocity (Fig 3B). In light of this finding, more studies are warranted to investigate the reductions of RTD during repeated contractions of maximal intensity and its link with changes in the overall performance.

A reduction of the nEMG_mean_ (-19.1 ± 15.4%; p<0.05) was observed between the first and last bouts in accordance with previous findings [25] whereas no changes in nEMG_peak_ were observed (-3.2 ± 19.5%; p = 0.71). A decrease of nRER (-21.1 ± 14.1%; p<0.001) was observed, indicating that fatigue was associated to changes in the EMG signal measured during the early part of the contraction (Fig 4A). Moreover, our results show that the decrease in nRER partly explain the reduction of EMG_mean_ values (R^2^ = 0.31; p<0.001; Fig 4B) whereas no associations were observed between changes in EMG_peak_ and nEMG_mean_ values (p = 0.43). These results suggest that the capacity of the central nervous system to maximally activate the muscle during the first milliseconds of the contraction was altered, whereas similar levels of maximal activation were reached later during the contraction. Because we could not record the EMG signals from the antagonist muscles (knee flexors), our results do not allow us to report changes in the co-activation levels between the agonist and antagonist muscles. It is possible that the reduction of the maximum and/or rapid knee torque production could be partly caused by an increase in the activation and force produced by the knee flexor muscles [26]. However, the contribution of this factor is probably limited as trained athletes usually display low levels of co-activation [27] and co-activation is usually reinforced to prevent hyper-extensions [28].

### Manifestation of peripheral fatigue after the repeated knee extensions

The reductions of resting potentiated twitch (eT_peak:_ -24.3 ± 5.3%, Fig 6) observed following the 160 maximal contractions completed by the subjects were comparable to those previously reported by Christian, Bishop, Billaut and Girard [2]. Similar levels of diminutions were observed for the evoked rate of torque development (eRTD: -24.7 ± 13.5%; Fig 6), suggesting a similar effect of alterations of the excitation-contraction coupling process on maximum force production and rapid-force production. No changes in M_max_ were observed at the end of the exercise protocol (Table 1), confirming the results from previous studies [6, 29] and suggesting that alterations of the excitation-contraction coupling were not due to changes in the sarcolemma excitability. Based on the reduction of Db_10:100_ observed following completion of the exercise protocol (-26.8 ± 6.2%, Table 1), impairments of the excitation-contraction coupling are likely to result from perturbations taking place beyond the muscle membrane [14]. Considering the blood lactate concentrations measured after the exercise (5.7 ± 4.0 mmol·L^-1^; p<0.001) and the plateau in O_2_ consumption (23.7 ± 6.4 mL·min^-1^·kg^-1^), repetition of maximal contractions performed at moderate velocity probably led a large accumulation of intracellular metabolites (ADP, P_i_ and H^+^). Large accumulation of these metabolites have been associated to impairments of the Ca^2+^ excitation-contraction coupling process [30] and alterations of the cyclical interaction of actin and myosin [31]. In light of the findings reported by Debold [31], reductions of RTD values could be due to an accumulation of H^+^ while decrease in peak torque production could be explained by an accumulation of P_i_.

### Manifestation of central fatigue after the repeated knee extensions

In line with previous findings [25], no changes in the voluntary activation (-4.3 ±1.6%, p = 0.08; Table 1) were observed after completion of the exercise protocol. The lack of changes in voluntary activation does not seem to be due to methodological points [32] since we used paired stimuli and there was no recovery period between the fatiguing protocol and the neuromuscular testing. The discrepancy between the results obtained for voluntary activation and nEMG_mean_ might be due to the fact that changes in nEMG_mean_ reflect both changes in rapid-activation (see reduction of nRER previously discussed) and maximal activation.

Results of the time-frequency analysis of the EMG signals revealed a substantial reduction of the mean power frequency (i*f*_mean_) during the first 225ms of the contraction (Fig 7). In line with previous findings, this result shows that repeated maximal contractions at moderate intensity may result into alterations of the recruitment and firing frequency of the motor units (MU) during the early part of the contraction [4, 8, 33, 34]. Indeed, supra-maximal firing rates, above the firing frequency needed to achieve maximum tetanic tension, are necessary to enhance RTD [8]. Moreover, the rapid force production has been recently strongly related to central parameters under fresh condition [35]. Especially, Johnson, Kipp, Norcross and Hoffman [35] showed that both the supraspinal drive and presynaptic inhibition can affect RTD. A number of spinal and supraspinal mechanisms have been suggested to influence the supraspinal drive and/or MU firing rate (for review see Gandevia [36]). Considering the high metabolic activity associated to the exercise protocol used in this study, it is likely that signals originated by the III/IV metabo-sensitive muscle afferents may have caused some inhibitions at the spinal and/or supraspinal levels [37, 38]. However, central fatigue can also come from more complex phenomenon like a loss of motivation [36] which is difficult to estimate. Finally, our results suggest that central fatigue might play an important role in the reduction of the firing frequency of the MUs during the first milliseconds of the contraction when subjects are repeating maximal contractions at moderate velocity.

## Conclusion

In summary, the performance decrease observed during repeated moderate velocity maximal exercise was critically associated to RTD decrease and to lesser extent to peak torque decrease. Moreover the fatigue etiology was different for each torque characteristic. Indeed, the maximum-torque capacity alteration seems to be strongly related to peripheral perturbations whereas rapid-torque capacity losses were associated to both central and peripheral fatigue. More particularly, the present study showed that repetition of maximal contractions at moderate velocity led to an alteration of the capacity of the central nervous system to maximally activate the muscle in the early milliseconds of the contraction. These results allow a better comprehension of the fatigue during repeated maximal exercise and especially in the course of the first milliseconds which is considered as a critical factor for performance and a determinant of injury risk.
